# Left atrial undifferentiated pleomorphic sarcoma causing left ventricular inflow tract obstruction: a case report

**DOI:** 10.1093/ehjcr/ytad560

**Published:** 2023-11-11

**Authors:** Guadalupe Baltazar Reyes-Robledo, Elias Noel Andrade-Cuellar, Ivan Alejandro Elizalde Uribe, Julieta D Morales-Portano

**Affiliations:** Department of Cardiology, National Medical Center November 20, Av. Felix Cuevas #540, Col. Del Valle Del. Benito Juarez, Mexico City, 03100 CP, Mexico; Department of Cardiology, National Medical Center November 20, Av. Felix Cuevas #540, Col. Del Valle Del. Benito Juarez, Mexico City, 03100 CP, Mexico; Department of Internal Medicine, National Medical Center November 20, Mexico City, Mexico; Department of Cardiology, National Medical Center November 20, Av. Felix Cuevas #540, Col. Del Valle Del. Benito Juarez, Mexico City, 03100 CP, Mexico

A 68-year-old woman with no relevant medical history presented a 2-month history of productive cough, several episodes of nocturnal paroxysmal dyspnoea, chest pain, and worsening dyspnoea over the last 48 h. The patient was evaluated and found to be alerted and oriented. Her vital signs showed a blood pressure of 130/90 mmHg, a heart rate of 116 b.p.m., and oxygen saturation of 88%. Bilateral disseminated lung crackles were heard, and a grade IV holosystolic murmur was detected at the mitral focus. The transthoracic echocardiogram revealed a mass in the left atrium attached to the posterior leaflet of the mitral valve, causing an obstruction of the left ventricular inflow tract. Transoesophageal echocardiogram (TEE) revealed a hyperechoic and heterogeneous image adhered to the posterior leaflet of the mitral valve, causing double mitral lesion, both severe (*Figure 1A, B, C*, Supplementary material online, *Videos S1* and *S2* of the Supplementary material). Systolic function of both ventricles was preserved, with an average transmitral gradient of 17 mmHg and a pulmonary systolic arterial pressure of 64 mmHg. Ventilatory support and intravenous diuretics were initiated, and urgent surgical intervention was planned.

**Figure 1:**
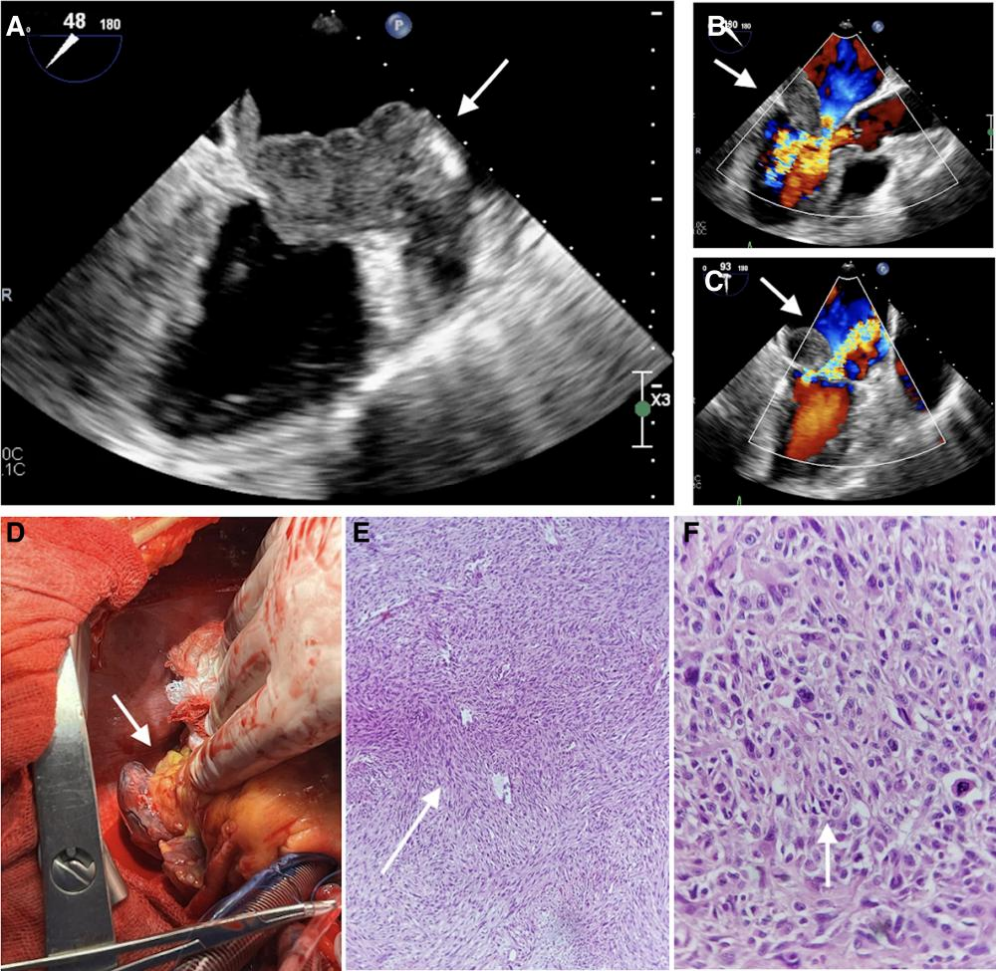
In the TEE with a 90° view, a hyper-echogenic, heterogeneous mass is observed, non-mobile, adhered to the entire posterior leaflet of the mitral valve, partially protruding through the valvular plane (*A*), resulting in severe double mitral lesions (*B* and *C*). (*D*) Macroscopic view of the tumour prior to surgical resection. (*E*) In immunohistochemistry at low magnification, the storiform pattern of the neoplasm is observed, with irregular and short intersecting fascicles, and marked cellularity (haematoxylin and eosin stain, 10 × magnification). (*F*) At higher magnification, we can appreciate the cellular detail. We observe pleomorphic, round, oval, and spindle-shaped cells, with varying sizes. Nuclei are round to elongated with irregular to indented contours and nucleoli. Some nuclei are lobulated and hyperchromatic, in addition to observing the presence of mitosis (haematoxylin and eosin stain, 40 × magnification). TEE, transoesophageal echocardiogram.

As part of the surgical protocol, a coronary angiography was performed, revealing circulation towards the tumour (see Supplementary material online, *Video S3* of the Supplementary material). Following a decision by the Heart Team, the patient underwent urgent tumour resection and mitral valve replacement. A tumour measuring 50 × 40 mm was observed, infiltrating, and extending to the free wall of the left ventricle (*Figure 1D*). Intracardiac resection was performed^1,2^, and intraoperative pathological analysis reported a malignant sarcoma tumour type. Subsequently, a 27 mm St. Jude mitral valve was implanted without complications.

The patient progressed without complications in the post-operative period in the coronary care unit. Follow-up echocardiography calculated a cardiac output of 4.2 L/min, a cardiac index of 2.3 L/min/m², a pulmonary capillary wedge pressure of 20 mmHg, a normally functioning mitral valve prosthesis, and no pericardial effusion. The histopathological analysis concluded an undifferentiated pleomorphic sarcoma (*Figure 1E* and *F*).

Radiation therapy and palliative chemotherapy were initiated^1^; however, 60 days after tumour resection, the patient was readmitted due to decompensation of heart failure. TEE revealed pre-prosthetic obstruction secondary to a 48 × 44 mm atrial mass, obstructing more than 50% of the left atrial outflow (see Supplementary material online, *Video S4* of the Supplementary material), resulting in a maximum velocity of 2.6 m/s and a mean prosthetic gradient of 14 mmHg. The decision was made to continue with supportive treatment and palliative measures.

## Supplementary Material

ytad560_Supplementary_Data

## Data Availability

The data underlying this article are available in the article and in its online Supplementary material.
